# First molecular subtyping and zoonotic significance of *Blastocystis* sp. in Dromedary (*C. dromedarius*) and Bactrian (*C. bactrianus*) camels in Iran: A molecular epidemiology and review of available literature

**DOI:** 10.1002/vms3.1442

**Published:** 2024-04-05

**Authors:** Ali Asghari, Amirhosein Yousefi, Roya Badali, Mohammad Reza Mohammadi, Laya Shamsi, Farajolah Maleki, Ali Mohammad Bahrami

**Affiliations:** ^1^ Zoonoses Research Center Ardabil University of Medical Sciences Ardabil Iran; ^2^ Department of Pharmaceutical Biotechnology University of Pavia Pavia Italy; ^3^ Department of Bacteriology Faculty of Medical Sciences Tarbiat Modares University Tehran Iran; ^4^ Faculty of Veterinary Medicine Department of Pathobiology, Urmia University Urmia Iran; ^5^ Clinical Research Development Unit Shahid Mostafa Khomeini Hospital, Ilam University of Medical Sciences Ilam Iran; ^6^ Faculty of Para‐Veterinary Medicine Ilam University Ilam Iran

**Keywords:** Ardabil, Iran, *Blastocystis* sp, camels, subtypes, zoonotic importance

## Abstract

**Background:**

*Blastocystis* sp. is a zoonotic protozoan parasite, and there is limited information about its molecular prevalence and subtypes (STs) distribution in camels globally, especially in Iran.

**Objectives:**

This study aimed to examine the prevalence, STs distribution, and zoonotic potential of *Blastocystis* sp. in one‐humped and two‐humped camels in Ardabil province, northwestern Iran.

**Methods:**

A PCR‐sequencing tool using the SSU rRNA gene was employed to examine the occurrence and genetic variation of *Blastocystis* sp. in 150 faecal samples from Bactrian (*Camelus bactrianus*, 50 samples) and Dromedary (*Camelus dromedarius*, 100 samples) camels in Ardabil province.

**Results:**

The overall prevalence of *Blastocystis* sp. in camels was determined to be 12% (18/150) through microscopy and PCR analyses. Phylogenetically, this study identified three distinct zoonotic STs: ST7, ST10, and ST14. ST10 was the most prevalent, comprising 50% (9/18) of the isolated STs from camels. ST14 closely followed with 38.9% (7/18), while ST7 made up 11.1% (2/18) of the total STs. In brief, ST10, ST14, and ST7 represented 50% (7/14), 35.7% (5/14), and 14.3% (2/14) of the *Blastocystis*‐positive cases in one‐humped camels, respectively. Further, each of the ST10 and ST14 accounted for 50% (2/4) of the *Blastocystis*‐positive samples in two‐humped camels. An analysis of the available data reveals that out of the 37–44 identified *Blastocystis* STs, 15 (ST1–ST7, ST10, ST14, ST15, ST21, ST24, ST25, ST26, and ST30) have been reported in camels. The predominant STs observed are ST10 and ST14. Furthermore, among the 15 zoonotic STs (ST1–ST10, ST12–ST14, ST16, and ST23) of *Blastocystis* reported thus far, nine zoonotic STs (ST1–ST7, ST10, and ST14) have been found in camels.

**Conclusions:**

These findings indicate that camels serve as a proper reservoir for a diverse array of *Blastocystis* STs and thereby can play a significant role in the transmission of this protozoan infection to humans, animals, and water reservoirs.

## Graphical abstract

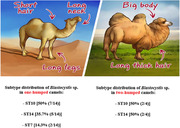

## 1 INTRODUCTION

*Blastocystis* sp., a single‐cell parasitic organism, is often present in the intestines of humans and various animal hosts (Asghari et al., 2020). The precise method of the transmission of *Blastocystis* is not fully understood, but it is believed to occur through the consumption of contaminated food or water (Asghari, Sadeghipour, et al., 2021). Inadequate sanitation and hygiene practices can increase the risk of infection. Although *Blastocystis* is commonly found in the human intestines, its role in causing symptoms is still a topic of debate (Asghari, Hassanipour, et al., 2021; Asghari, Sadrebazzaz, et al., 2021). Some studies have suggested a connection between *Blastocystis* infection and gastrointestinal symptoms such as diarrhoea, abdominal pain, and bloating (Liu et al., 2023). However, other studies have failed to establish a significant association between the presence of this protozoan and symptoms (Asghari, Hassanipour, et al., 2021).

It is estimated that a significant portion of the population may carry *Blastocystis* without showing any symptoms (Asghari, Yousefi, et al., 2024; Shams et al., 2024). This implies that factors apart from the presence of the parasite might contribute to symptom development in certain individuals (Sheikh et al., 2020; Shams, Asghari, et al., 2022). Host factors, such as the immune response and gut microbiota composition, are believed to influence whether an individual develops symptoms or remains asymptomatic (Naguib et al., 2022; Qi et al., 2023). Treatment options for *Blastocystis* infection include antibiotics, antiprotozoal drugs, and probiotics. However, the decision to treat the infection is often based on the presence of symptoms and the severity of the infection (Asghari et al., 2019; Rauff‐Adedotun et al., 2023). Asymptomatic carriers may not require treatment unless they are at the risk of transmitting the parasite to others or if the infection is causing complications (Shams, Shamsi et al., 2022; Tantrawatpan et al., 2023). *Blastocystis* can be transmitted through human‐to‐human, animal‐to‐human, animal‐to‐animal, and human‐to‐animal contact (Asghari, Banavand, et al., 2024). The extent to which non‐human hosts can act as reservoirs for *Blastocystis* subtypes (STs) that infect humans remain unclear. The presence of similar *Blastocystis* STs in both humans and animals has led to the hypothesis that the organism has the potential to be zoonotic (Ali et al., 2022; Deng et al., 2023).

The molecular analysis of *Blastocystis*‐specific small subunit ribosomal RNA genes (SSU rRNA) has been the primary method for subtyping *Blastocystis* for over 15 years. Instead of using traditional binomial species names, *Blastocystis* terminology now relies on assigning ST numbers to differentiate the organism. *Blastocystis* has been classified into nearly 44 STs, of which at least 37 have been confirmed as valid in humans and/or various non‐human hosts worldwide (Santin et al., 2024). ST1–ST10, ST12‐ST14, ST16, and ST23 are commonly found in both humans and animals, while the remaining STs are typically associated with non‐human hosts (Jiménez et al., 2022). However, there have been occasional reports of infections in humans (Mahdavi et al., 2022; Shams et al., 2021; Yang et al., 2021).

One‐hump camels, commonly referred to as dromedaries (*C. dromedarius*), and two‐hump camels, known as Bactrian camels (*C. bactrianus*), are significant creatures possessing distinct qualities that render them invaluable to human communities. Dromedaries are well suited for arid habitats and serve as means of transportation, providers of milk, meat, and hides in desert regions. Bactrian camels, on the other hand, have adapted to thrive in extremely cold temperatures with limited food resources and are also utilized for transportation, milk, meat, and hides in Central Asia (Sazmand et al., 2019; Yang et al., 2021).

Although *Blastocystis* infects various animals globally, including mammals, birds, reptiles, and amphibians, there is a significant lack of testing and ST characterization for this parasite. Most studies have focused on its impact on human health, with limited investigation into its presence and potential effects in animals, particularly camels. Therefore, this study aimed to examine the prevalence, distribution of STs, and zoonotic potential of *Blastocystis* sp. in one‐humped and two‐humped camels in Ardabil, northwestern Iran.

## 2 MATERIALS AND METHODS

### 2.1 Sampling

**FIGURE 1:**
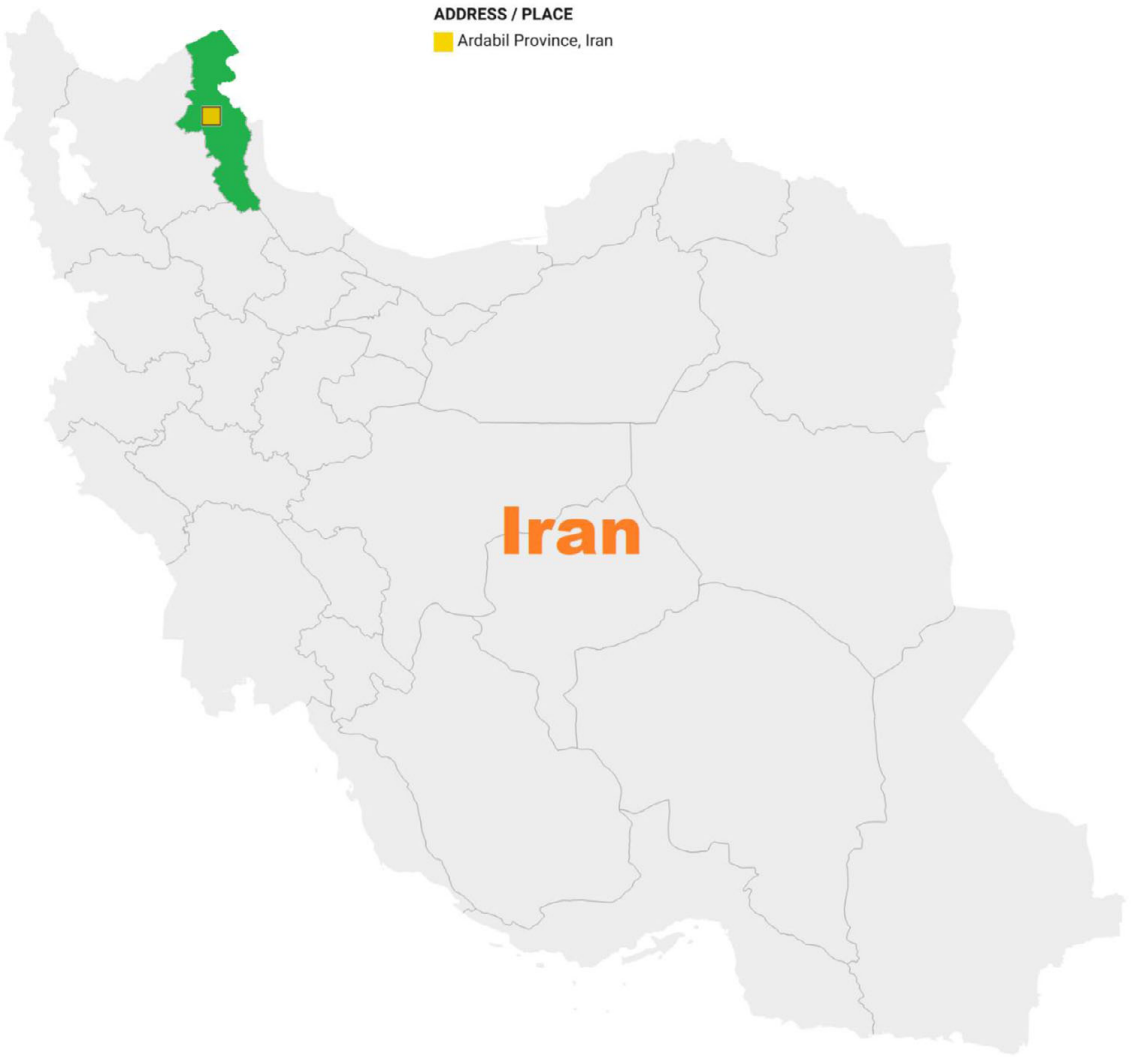
Map of Iran and sampling location of camels (Ardabil province).

Between July 2021 and October 2022, a total of 150 faecal samples were collected from Bactrian (*C. bactrianus*, 50 samples) and Dromedary (*C. dromedarius*, 100 samples) camels in Ardabil province, northwestern Iran (Figure 1). Out of the 150 samples, 21 were collected from camels aged less than 2 years, 22 from aged between 2 and 6 years, and 107 from aged over 6 years. All animals that were examined were found to be in good health. There were no observable signs of illness or gastrointestinal problems, and their faecal consistency was normal. The samples were individually placed in labeled bags containing basic information. They were promptly transported to the parasitology laboratory at Shiraz University of Medical Sciences while maintaining a cool temperature. Upon arrival, the samples were stored in 2.5% potassium dichromate at a temperature of 4°C, ensuring their preservation for future study.

### 2.2 Microscopy‐based detection and genomic DNA extraction

First, the samples were examined by direct microscopy using both saline and iodine wet mount preparation. Faecal smears were also prepared and were stained with Wheatley's Trichrome stain. The genomic DNA (gDNA) was extracted from the faecal samples using the E.Z.N.A. stool DNA kit from Omega (Norcross) after washing each sample three times with distilled water to remove potassium dichromate. Approximately 300 mg of faeces from each sample was used for the extraction, following the manufacturer's guidelines. Subsequently, the extracted gDNA samples were stored at −20°C.

### 2.3 PCR amplification

The gDNA samples were analysed by PCR amplification of ∼600 bp of the SSU rRNA gene of *Blastocystis* sp. using the primers RD5 (5′‐ATCTGGTTGATCCTGCCAGT‐3′) and BhRDr (5′GAGCTTTTTAACTGCAACAACG‐3′) to identify the presence of *Blastocystis* sp. Infection (Scicluna et al., 2006). All PCR reactions were performed in a final volume of 25 µL. Each reaction included 1 µL (10 pm) of each primer, 12.5 µL of 1 × Taq DNA Polymerase Master Mix RED from Ampliqon, 7.5 µL of distilled water, and 3 µL of extracted DNA. Positive controls (DNA of a human‐derived *Blastocystis* sp. isolate) and negative controls (distilled water) were included in all PCR amplification reactions. Finally, the PCR products were visualized by electrophoresis on a 1.5% agarose gel stained with a safe stain (SinaClon).

### 2.4 Sequencing and phylogenetic analysis

The DNA fragments obtained from an agarose gel were purified using the GeneJet Gel Extraction Kit from Thermo Fisher Scientific Inc. Subsequently, bidirectional sequencing was performed using the same PCR primers on a Genetic Analyser ABI 3130 XL from Macrogen. The resulting sequencing chromatograms were edited and aligned using the Geneious Prime 2020.0.3 software to generate a single SSU rRNA consensus sequence for each sample. To detect mixed infections, the chromatograms were examined for any double or ambiguous nucleotide peaks. The obtained nucleotide sequences were then compared with reference sequences in GenBank through BLAST analyses to determine the *Blastocystis* STs. Finally, all the nucleotide sequences generated in this study were deposited in the GenBank Data Library under accession numbers OR755854‐OR755858, OR755879‐OR755881, OR755883‐OR755886, and OR757108‐OR757113. Phylogenetic reconstruction of *Blastocystis* sp. was conducted using the neighbour‐joining (NJ) method, with bootstrap values (1000 replicates), in the MEGA X software. *Blastocystis lapemi* served as the outgroup for the phylogenetic analyses.

### 2.5 Statistical analysis

The data collected were analysed using the SPSS software (version 20, IBM Inc.). The chi‐square test was utilized to establish the correlation between *Blastocystis* infection and age groups. A significance level of *p* < 0.05 was considered statistically significant.

## 3 RESULTS

### 3.1 Occurrence of *Blastocystis* sp. in camels

The overall prevalence of *Blastocystis* sp. in camels was determined to be 12% (18/150) through microscopy and PCR analyses. The microscopic findings aligned with the PCR results. In brief, *Blastocystis* was detected in 14% (14/100) and 8% (4/50) of one‐humped (*C. dromedarius*) and two‐humped (*C. bactrianus*) camels, respectively.

### 3.2 STs distribution and phylogenetic analysis of *Blastocystis* sp. in camels

**FIGURE 2:**
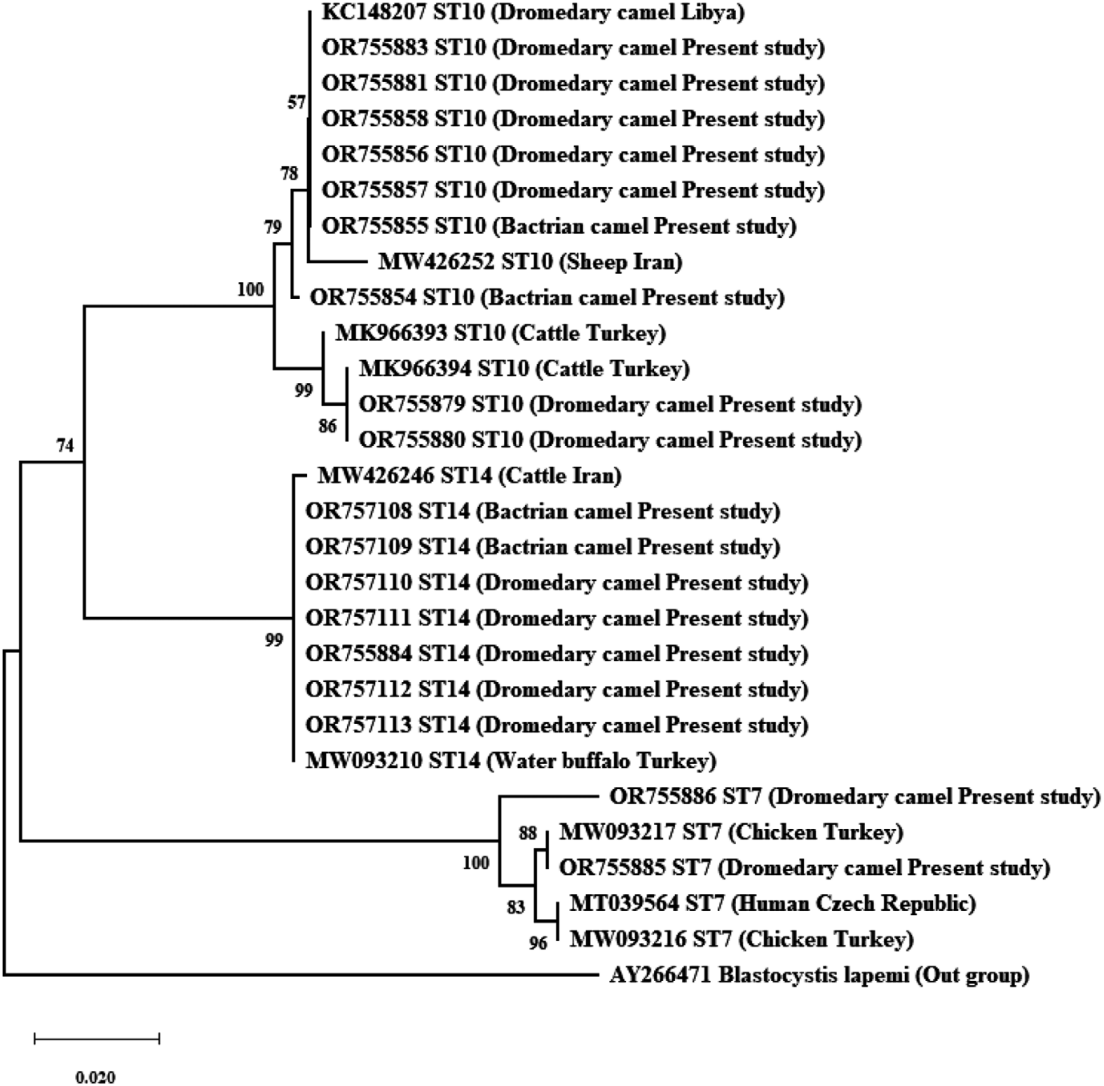
The phylogenetic tree was built using the neighbour‐joining method and sequences obtained from GenBank. The percentage of replicate trees, in which the associated taxa clustered together in the bootstrap test (1000 replicates), is indicated next to the branches.

All 18 *Blastocystis*‐positive samples were successfully amplified and subtyped by analysing the sequence of the SSU rRNA gene. The sequence electropherograms showed no double or ambiguous peaks. By conducting a BLAST search and performing sequence analysis of the SSU rRNA gene, three zoonotic STs were recognized: ST7, ST10, and ST14. Overall, ST10 (50% [9/18]) was the most common ST isolated from camels in the present study, followed by ST14 (38.9% [7/18]) and ST7 (11.1% [2/18]). In brief, ST10, ST14, and ST7 represented 50% (7/14), 35.7% (5/14), and 14.3% (2/14) of *Blastocystis*‐positive cases in one‐humped camels, respectively. Additionally, each of the ST10 and ST14 accounted for 50% (2/4) of *Blastocystis*‐positive samples in two‐humped camels. Age groups, prevalence, and STs distribution of *Blastocystis* sp. in camels of the present study are shown in Table 1. Furthermore, the frequency and STs distribution of *Blastocystis* sp. in this study were compared to previous investigations on camels based on the available data (Table 2). Phylogenetic analysis demonstrated that all camel sequences were closely (99%–100%) related to sequences from animals or humans in GenBank, and ST‐based clustering was observed in the NJ tree (Figure 2).

**TABLE 1:** Prevalence and subtypes (STs) distribution of *Blastocystis* sp. in camels based on age groups.

Age (years)	No. of examined camels	No. of infected camels (%)	STs (No.)	p‐Value
<2	21	3 (14.3)	ST10 (2), ST14 (1)	>0.05
2–6	22	3 (13.6)	ST10 (1), ST14 (2)	
>6	107	12 (11.2)	ST7 (2), ST10 (6), ST14 (4)	
Total	150	18 (12)	ST7 (2), ST10 (9), ST14 (7)	

**TABLE 2:** The global distribution of *Blastocystis* STs in camels.

				Subtyping of infected samples^a^		
Author, year	Countries	Total samples (no.)	Infected samples (%)	Subtyped^b^ (no/%)	Unidentified^c^ (no/%)	Zoonotic STs^d^ (no/%)
Yang et al., 2021	China	638	139 (21.8)	ST10 (49/35.2), ST14 (21/15.1), ST30 (19/16.7), ST24 (14/10.1), ST25 (8/5.7), ST21 (1/0.7), ST26 (1/0.7), novel ST (21/15.1)	5/3.6	70/50.3
Zhang et al., 2021	China	40	14 (35)	ST10 (7/50), ST2 (6/42.8), ST14 (1/7.2)	‐	14/100
Mokhtar & Youssef, 2018	Egypt	20	5 (25)	ST1 (3/60), Mixed STs^e^ (1/20)	1/20	4/80
Zhao et al., 2017	China	10	5 (50)	ST10 (4/80), ST1 (1/20)	‐	5/100
Alfellani et al., 2013	Libya	196	47 (24)	ST5 (20/42.5), Mixed STs^f^ (7/14.9), ST10 (6/12.8), ST1 (5/10.6), ST3 (5/10.6), ST14 (3/6.4), ST15 (1/2.2)	‐	39/83
Asghari et al., 2023	Iran	150	18 (12)	ST10 (9/50), ST14 (7/38.9), ST7 (2/11.1)	‐	18/100

^a^ Out of the positive samples of *Blastocystis*.

^b^ Some have been subtyped

^c^ But some have not subtyped or not determined

^d^ The number and percentage of zoonotic subtypes are computed from ST1–ST10, ST12‐ST14, ST16, and ST23.

^e^ Mix infections included ST1, ST4, and ST6.

^f^ Has not been mentioned.

## 4 DISCUSSION

*Blastocystis* sp. is a protozoan parasite that can be found in the intestines of humans and animals such as camels. Animals can serve as a possible reservoir for human infection because of the poor host specificity and zoonotic potential of *Blastocystis* sp. (Higuera et al., 2023).

Studying and understanding the presence and impact of *Blastocystis* STs and other intestinal parasites in camels are important for several reasons. First, camels are vital animals in many regions, particularly in arid and desert areas, where they are used for transportation, milk production, and meat. Therefore, the health and well‐being of camels are crucial for the livelihoods and economies of these regions. Intestinal parasites, including *Blastocystis* sp., can cause health issues in camels, such as diarrhoea, weight loss, and reduced productivity. By understanding the prevalence and effects of these parasites, appropriate preventive and control measures can be implemented to ensure the overall health and productivity of camel populations. Second, camels can act as carriers of parasites that can infect humans and other animals. *Blastocystis* sp. has been found to infect humans and is considered a potential zoonotic parasite, meaning it can be transmitted between animals and humans. Understanding the prevalence and transmission dynamics of *Blastocystis* sp. in camels is important for assessing the risk of human infection and implementing measures to prevent its spread. Additionally, studying *Blastocystis* sp. and other intestinal parasites in camels can contribute to our knowledge of parasite ecology and evolution. It can help us understand the life cycle, transmission patterns, and genetic diversity of these parasites, which can have implications for their control and management (Abubakr et al., 2000; Birhanu et al., 2014; Bouragba et al., 2020; Sazmand & Joachim, 2017; Sazmand et al., 2019).

The current study, being the first conducted in Iran, examined the prevalence, STs distribution, and zoonotic significance of *Blastocystis* infection in one‐humped (*C. dromedarius*) and two‐humped (*C. bactrianus*) camels in Ardabil province, located in northwestern Iran. The overall prevalence of *Blastocystis* in camels examined in this study was 12% (18/150), which is relatively low compared to studies conducted in China (21.8%–50%) (Yang et al., 2021; Zhao et al., 2017; Zhang et al., 2021), Egypt (25%) (Mokhtar & Youssef, 2018), and Libya (24%) (Alfellani et al., 2013). This variation may be attributed to geographical differences, health conditions, animal rearing and feeding practices, as well as the sensitivity of diagnostic methods used. Moreover, the current study found a higher incidence of *Blastocystis* infection in one‐humped camels (14%) compared to two‐humped camels (8%), which may be attributed to the sample size and their living conditions. Meanwhile, consistent with a previous study (Yang et al., 2021), *Blastocystis* was detected in camels across all three age groups investigated. The infection rates were 14.3% (3/21), 13.6% (3/22), and 11.2% (12/107) for animals aged <2 years, 2−6 years, and >6 years, respectively (*p* > 0.05). These findings suggest a lower infection rate in older animals (>6 years) when compared to younger age groups (<2 years and 2−6 years). However, contrasting findings were observed in the colonization of *Blastocystis* in cattle. It was mentioned that age may play a role in influencing prevalence, as younger animals were found to have a lower prevalence compared to older animals (Hublin et al., 2021).

Phylogenetic investigations revealed that the camels in this study were infected with three different zoonotic STs: ST7, ST10, and ST14. ST10 and ST14 are commonly found in camels and ruminants, as observed in previous studies (Mokhtar & Youssef, 2018; Shams et al., 2021; Yang et al., 2021; Zhao et al., 2017; Zhang et al., 2021). However, the presence of ST7 in camels, which is primarily associated with bird infections, suggests that water and food consumed by camels may be contaminated with bird or human faeces. Overall, there is limited global research on the prevalence and subtyping of *Blastocystis* in camels (Alfellani et al., 2013; Mokhtar & Youssef, 2018; Yang et al., 2021; Zhao et al., 2017; Zhang et al., 2021). However, an analysis of the available data reveals that out of the 37–44 identified *Blastocystis* STs, 15 (ST1–ST7, ST10, ST14, ST15, ST21, ST24, ST25, ST26, and ST30) have been reported in camels. The predominant STs observed are ST10 and ST14. Furthermore, among the 15 zoonotic STs (ST1–ST10, ST12–ST14, ST16, and ST23) of *Blastocystis* reported thus far (Jiménez et al., 2022; Santin et al., 2024; Shams et al., 2024), nine zoonotic STs (ST1–ST7, ST10, and ST14) have been found in camels. These findings indicate that camels serve as a proper reservoir for a diverse array of *Blastocystis* STs and thereby can play a significant role in the transmission of this protozoan infection to humans, animals, and water reservoirs.

## 5 CONCLUSION

Based on the present study and previous investigations, it has been determined that camels can serve as a suitable reservoir for *Blastocystis* infection and its related STs. This highlights their possible role in transmitting zoonotic STs to humans and other animals. However, further extensive and detailed research is necessary to ascertain the specific and non‐specific range of *Blastocystis* STs in camels.

## AUTHOR CONTRIBUTIONS

Ali Asghari planned and designed the study. Ali Asghari, Amirhosein Yousefi, Roya Badali, Farajolah Maleki, and Ali Mohammad Bahrami were involved in sample collection and methodology. Ali Asghari, Mohammad Reza Mohammadi, and Laya Shamsi conducted the molecular analysis. Ali Asghari wrote the manuscript and revised it. All authors have read and approved the final manuscript.

## CONFLICT OF INTEREST STATEMENT

The authors declare no conflicts of interest.

## FUNDING INFORMATION

This study did not receive funding.

## ETHICS STATEMENT

The sampling stages and their evaluation were reviewed and approved by the ethics committee of Shiraz University of Medical Sciences in Fars province, southwest Iran. Animal sampling was conducted without causing harm and with the owners' consent.

## Data Availability

The datasets used and/or analysed during the current study are available in the online version.
